# Prevalence of Depression and Its Impact on Quality of Life in Cancer Survivors: An Observational Study From South India

**DOI:** 10.7759/cureus.100280

**Published:** 2025-12-28

**Authors:** Ramya Lakshmi, Maanushree Arunkumar, M Harshidha, Mahalakshmi Arunachalam, Ardhanaari M, M Manickavasagam, Ravi Chandran Ambalathandi

**Affiliations:** 1 Medical Oncology, Sri Ramachandra Institute of Higher Education and Research, Chennai, IND; 2 Applied Psychology, Sri Ramachandra Institute of Higher Education and Research, Chennai, IND; 3 Psychiatry, Meenkashi Medical College, Chennai, IND; 4 Medical Oncology, Saveetha Medical College, Chennai, IND

**Keywords:** cancer survivors, depression prevention, ham-d, quality of life, whoqol-bref

## Abstract

Background: Advances in cancer therapy have extended survival, shifting focus toward survivorship and quality of life (QoL). Depression is a common but under-recognized issue among cancer survivors, influencing psychological adaptation and well-being.

Aim: To assess the prevalence of depression and its impact on quality of life among cancer survivors beyond one year of diagnosis.

Methods: A cross-sectional study was conducted among 55 cancer survivors at a tertiary care centre. Depression was assessed using the Hamilton Depression Rating Scale (HAM-D), and QoL was evaluated with the WHOQOL-BREF questionnaire (covering physical, psychological, social, and environmental domains).

Results: The mean age of the study participants was 55.7 ± 11.5 years; 15 (27.3%) were male and 40 (72.7%) female. Depression was observed in 42 survivors (76%), of whom 22 (52.3%) had mild depression, 14 (33.3%) had moderate depression, and 6 (14.2%) had severe depression. The mean WHOQOL-BREF scores were 55.7 in the physical domain, 54.6 in the psychological domain, 52.1 in the social domain, and 77.5 in the environmental domain. Quality of life declined progressively with increasing severity of depression, with individuals experiencing severe depression reporting the lowest scores across domains.

Conclusion: Depression is highly prevalent among cancer survivors (76%) and strongly associated with impaired QoL, especially in environmental (Mean score:77.5) and physical domains (Mean score:55.7). Routine psychosocial screening and integrative supportive interventions are essential to optimize survivorship outcomes.

## Introduction

Among all types of disease, cancer remains a leading cause of mortality and morbidity globally, accounting for approximately 10 million deaths annually [1]. Depression and anxiety are the most common psychological issues among patients with different types of cancer [2], even in the general population, with an estimated prevalence of 3.6% of the global population [3]. However, the rate of depression in cancer patients is three times higher compared with the general population [4]. Beyond biological and clinical factors, the psychosocial determinants, such as depression and anxiety, are a major cause for poor survival outcomes [5]. Depression affects up to 20-30% of cancer patients, manifesting through persistent sadness, fatigue, loss of motivation, and reduced treatment adherence [6]. With the affected individuals, the type of treatment and symptoms may vary; however, the sense of uncertainty, sadness, anxiety, and depressive status still exist [7]. These nonpathological depressive and anxiety disorders may lead to increased levels of hopelessness, other psychiatric syndromes, and sometimes increased risk of suicide among the cancer patients [2].

Furthermore, the depression of cancer patients leads to a poorer quality of life (QOL), affects the patient outcomes, and a higher rate of mortality was also observed with depression in cancer [8]. A meta-analysis, which included 25 independent studies, revealed that minor and/or major depression increases mortality rates by up to 39%, and that patients displaying even a few depressive symptoms may be at a 25% increased risk of mortality [9]. Therefore, the proper diagnosis of depression and anxiety among the affected patients who are undergoing clinical treatment with a proper diagnostic tool, such as a good validated questionnaire, may improve the individual's quality of life and further increase the patient's survival. Therefore, in the present study, we aimed to identify the depression status and QoL of different types of cancer-affected individuals in the south Indian populations by using the Hamilton Depression Rating Scale (HDRS-17) and World Health Organization Quality of Life-Brief Version (WHOQOL-BREF) questionnaire in a tertiary care hospital at Sri Ramachandra Institute of Higher Education and Research, Chennai.

## Materials and methods

Study population

The present study included a total of 55 cancer patients, of whom 15 (27.3%) were male, and 40 (72.7%) female, who were included between June 2025 and August 2025, and this study was conducted at the Department of Medical Oncology, Sri Ramachandra Institute of Higher Education and Research (SRIHER), Chennai, Tamil Nadu, India. The Dravidian population, comprising individuals from the states of Tamil Nadu, Andhra Pradesh, Karnataka, Kerala, Telangana, and Puducherry, who visited SRIHER, was included in this study. With the prior approval of the Institutional Ethics Committee (CSP-MED/25/NOV/122/296) of SRIHER and proper written informed consent, the study participants were recruited for this study. The patients aged <18 years, who are unable to manage pain and carry out self-care activities, were excluded from this study. Further, participants who cannot answer the questions correctly due to severe illnesses, psychiatric disorders, or neurological illnesses were also excluded from this study.

Data collection and assessment tools

The clinician-rated Hamilton Depression Rating Scale (HDRS-17) was used to evaluate the depressive severity of cancer patients, which considers 17 items scored between 0 and 4 points [10]. Each item is scored from 0 to 3 depending on the severity of the depressive symptoms, with cut-off points for depressed mood ≥8 points. The scoring ranges are 0-7 for depressive symptoms, 8-16 for mild depression, 17-23 for moderate depression, and over 24 for severe depression. Further, the participants' personality test was also completed with the self-reporting Barratt Impulsiveness Scale (BIS-11) [11], prior to HRDS-17 questionnaire collection. In addition to the HDRS-17 questionnaire, the clinical and demographic characteristics were collected from the patient's medical record sheet, and lifestyle characteristics were also collected alongside. Furthermore, the quality of life (QoL) was assessed using the WHOQOL-BREF questionnaire, which measures four domains: physical health, psychological health, social relationships, and environmental well-being [12]. Each questionnaire was scored as per standardized scoring instructions provided for each item. The overall depression and QoL scores were calculated for every patient. Sub-analysis was subsequently performed to compare QoL among patients with and without depression, and across varying depression severity categories (mild, moderate, severe).

Statistical analysis

The collected data were organized in a Microsoft Excel spreadsheet, followed by analysis using R software version 4.5.2. (R Core Team, 2024) Continuous variables were represented as the mean ± standard deviation, while categorical data were shown as frequencies and percentages. To explore the correlation between depression severity and QoL scores, mean comparison and trend analysis were utilized. A p-value below 0.05 was considered statistically significant.

## Results

A total of 55 different types of cancer participants were assessed for depressive severity and quality of life. The mean age of the study participants was 55.7 ± 11.5 years, representing a middle-aged to older adult population. The gender distribution demonstrated a predominance of females (40, 72.7%) compared with males (15, 27.3%). Among lung cancer patients, males were more commonly affected (8, 53.3%), whereas breast cancer occurred exclusively in females, accounting for 18 cases (45.0%). The other studied characteristics of participants were documented in Table 1. 

**Table 1:** Distribution of patients' demographic characteristics.

Characteristics	Category	Number of patients N=55 (%)
Age Group (Yrs)	< = 50	18 (32.7)
	51 - 60	16 (29.1)
	>60	21 (38.2)
Gender	Male	15 (27.3)
	Female	40 (72.7)
Education	Graduate	24 (43.6)
	School education	20 (36.3)
	Illiterate	11 (20.0)
Occupation	Employed	38 (69.0)
	Unemployed	17 (30.9)
Marital status	Married	48 (82.7)
	Unmarried	2 (3.6)
	Widowed	5 (9.0)
Types of cancer: Male	Lung cancer	8 (53.3)
	Colon cancer	3 (20.0)
	Rectal cancer	4 (26.6)
Female	Breast cancer	18 (45.0)
	Ovarian cancer	13 (32.5)
	Endometrial cancer	9 (22.5)

**Figure 1:**
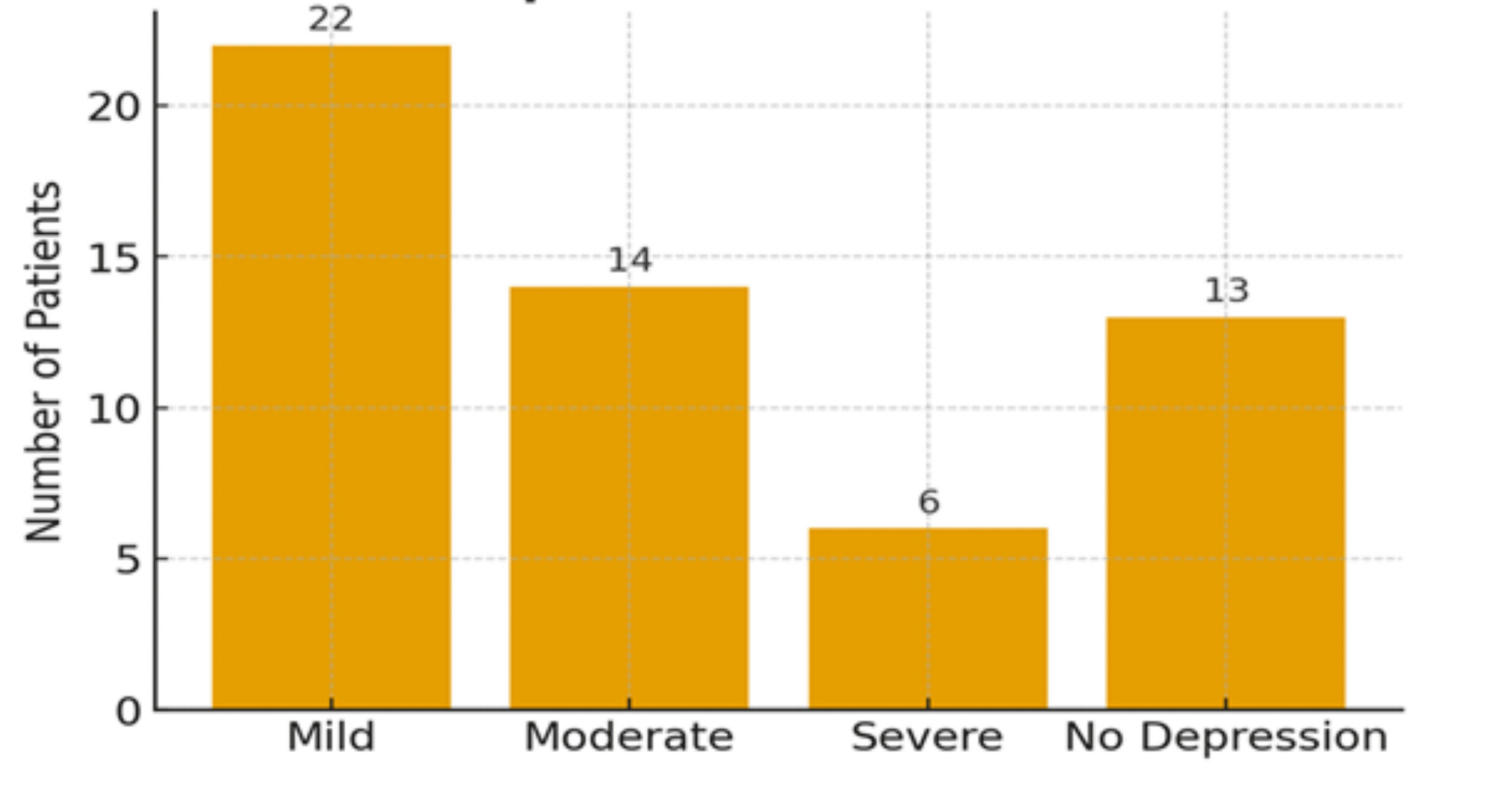
Depressive analysis of the studied cancer patients.

With respect to depressive symptoms, out of 55 patients, 22 (40.0%) patients experienced mild depression, 14 (25.4%) reported moderate depression, and 6 (10.9%) had severe depressive symptoms, while 13 (23.6%) were not depressed, as shown in Figure 1. 

**Figure 2:**
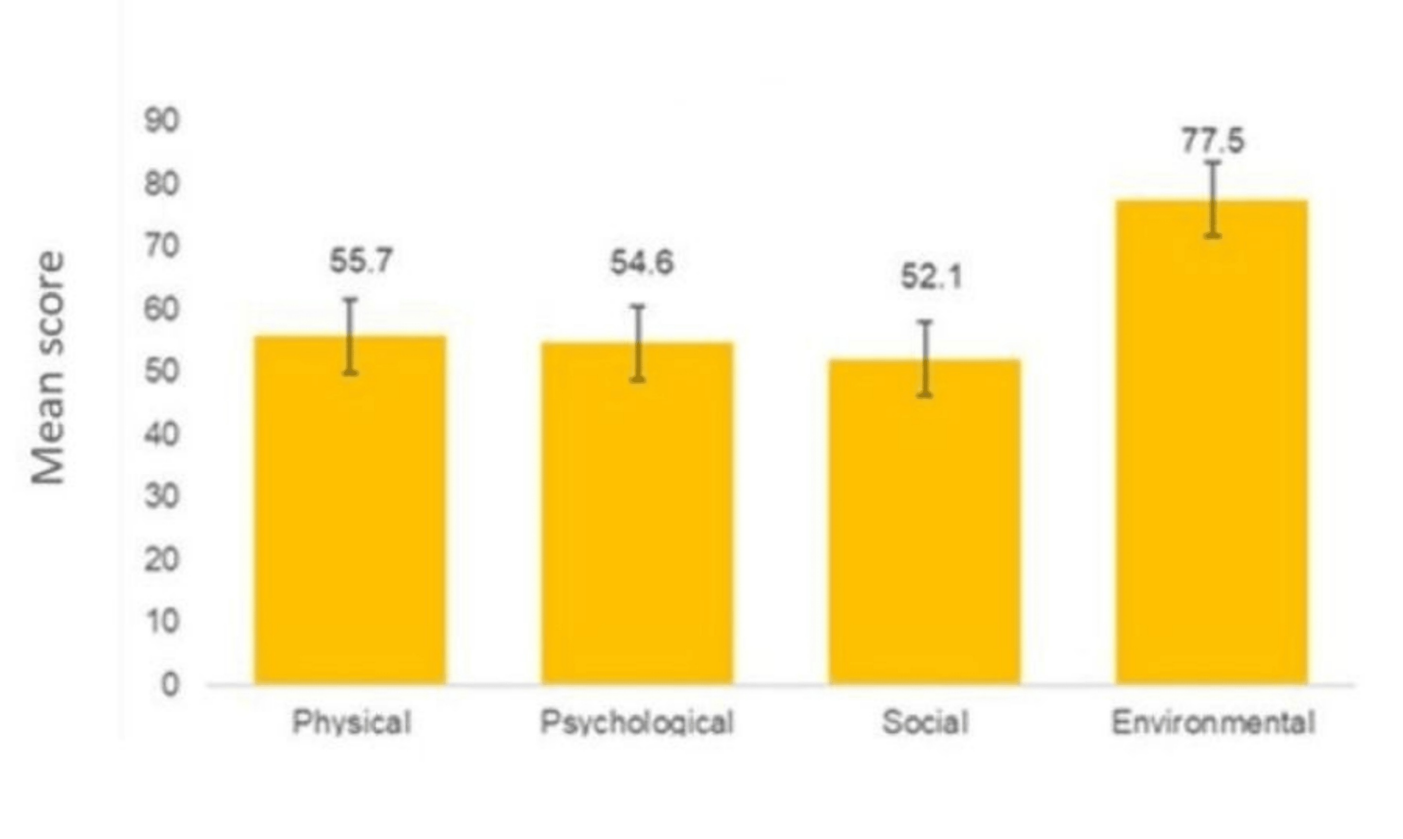
The WHOQOL-BREF analysis of the studied participants.

Quality-of-life (QoL) analysis across the four WHOQOL-BREF domains revealed that the environmental domain contributed the highest mean score with 77.5, reflecting better satisfaction with aspects such as safety and living conditions. Social relationships scored the lowest, 52.1, indicating significant impairment in interpersonal communication and emotional expression, with many participants reporting social withdrawal and isolation due to stigma surrounding cancer (Figure 2).

**Figure 3:**
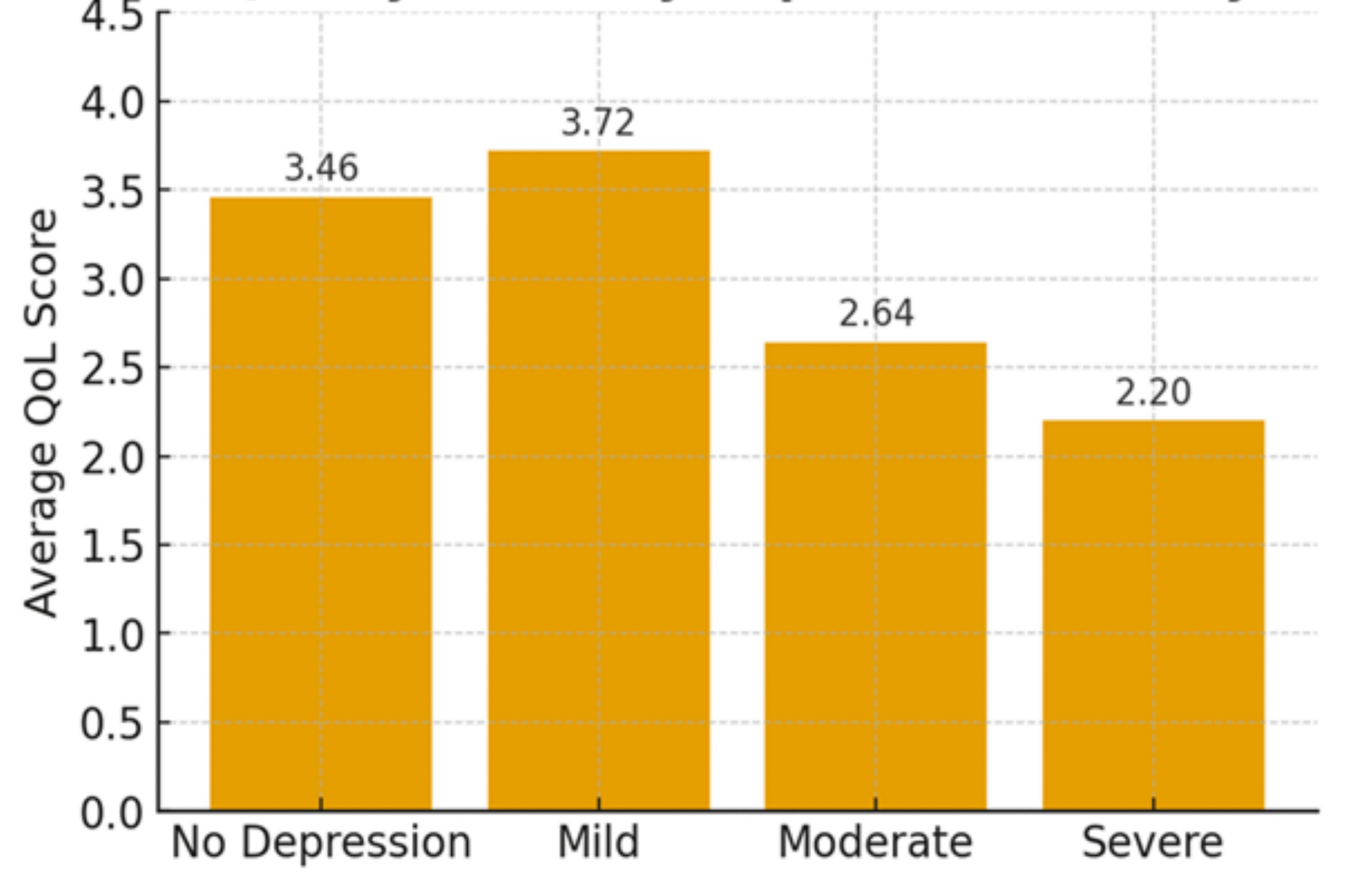
The overall QoL depression severity analysis.

The assessment of quality of life (QoL) among oncology patients revealed distinct variations across different levels of depression. Patients with mild depression reported the highest average QoL score (M=3.72), even surpassing those without depression (M=3.46). In contrast, patients with severe depression demonstrated the lowest average QoL score (M=2.20), indicating a significant impact of depressive symptoms on overall well-being. Those with moderate depression also showed a marked reduction in QoL (M=2.64), with values closely aligned to those of the severe group, suggesting that both moderate and severe depression considerably impair quality of life. These findings underscore the negative association between the severity of depression and the quality of life in oncology patients (Figure 3).

The relatively higher QoL observed in patients with mild depression may represent a state of preserved psychological insight and adaptive coping, which can positively influence perceived well-being and engagement with care. Conversely, the group without depression may include individuals with unrecognized or subclinical distress that is not captured by screening instruments but still impacts QoL. Importantly, the progressive decline in QoL with increasing depression severity in our study supports a clear inverse relationship between depressive burden and quality of life in oncology patients.

## Discussion

The present study was conducted among patients with various types of cancer receiving active treatment at a tertiary care institution, SRIHER, Chennai, Tamil Nadu. Health‑care providers are facing the great challenge to treat cancer patients, due to physical and psychological problems, which mainly include depression. In our investigation, the prevalence of depression among the studied patients was found to be 10.9% of severe depression. The prevalence of depression observed in the present study was substantially higher than that reported in a recent 2025 study by Soni Ray in Bihar, which identified a depression rate of 3.2% among healthy individuals [13]. In contrast, a study conducted at a tertiary care hospital in Kerala reported a depression prevalence of 21.5% among breast cancer patients receiving active treatment [14]. Similarly, a study from Malaysia involving breast cancer patients reported a depression prevalence of 22% [15]. The depression rate observed in the present study was lower in comparison with the studies conducted in Malaysia and Bihar, which focused exclusively on cancer patients. In contrast, the present study included patients with different types of cancer, and the sample size was relatively small, which may have influenced the observed prevalence. Furthermore, a recent meta-analysis encompassing 65 cohort studies reported a significant association between depression and cancer outcomes, with a hazard ratio of 1.83 (95% CI: 1.47-2.28), indicating an increased mortality risk among cancer patients with depression [16].

Despite the well-documented effects of depression on quality of life, its impact on cancer progression and mortality remains inconclusive. However, the clinical relevance of depression following a cancer diagnosis and during active treatment is well established [2]. The previous cross-sectional studies reported that depression is inversely related to the quality of life among breast cancer patients and survivors [17,18]. In our study, QoL analysis across the four WHOQOL-BREF domains revealed that the environmental domain contributed the highest mean score, reflecting better satisfaction with aspects such as safety and living conditions. Social relationships scored the lowest, indicating significant impairment in interpersonal communication and emotional expression, with many participants reporting social withdrawal and isolation due to stigma surrounding cancer. Similarly, a study from the surgical oncology inpatient wards of a tertiary care hospital in Kancheepuram, Tamil Nadu, also reported that depression was associated with social factors and directly affects the QoL of breast cancer patients [19]. Another study from the South Indian population revealed that patients with depression were poorly associated with overall QoL than those without depression [14]. Further, the study of Chinese breast cancer reported that QoL was significantly and independently associated with a poorer level of QoL [20,21].

Limitations

The present study has certain limitations. First, the sample size was too small, with only 55 cancer patients from South India, and the study participants may not be representative as per convenience sampling. Second, the long-term effect of the psycho‑oncology program intervention was not assessed, as it is a partial fulfilment of the Post Graduate program. Importantly, the univariate or multivariate analysis was not performed due to the small sample size, and the study focuses on depression and QoL intervention rather than risk factors. Despite these limitations, our study observed that mild to severe depression is linked to the quality of life of the cancer survivors.

## Conclusions

In the population studied, depression ranging from mild to severe was noted and linked to quality of life. Among the domains assessed, the environmental domain had the highest average score compared to social relationships and psychological health. To validate our findings, further research with a larger, multi-center sample and both univariate and multivariate analyses is necessary.
